# Daratumumab Improves Depth of Response and Progression-free Survival in Transplant-ineligible, High-risk, Newly Diagnosed Multiple Myeloma

**DOI:** 10.1093/oncolo/oyac067

**Published:** 2022-04-24

**Authors:** Andrzej J Jakubowiak, Shaji Kumar, Rohan Medhekar, Huiling Pei, Patrick Lefebvre, Shuchita Kaila, Jianming He, Marie-Hélène Lafeuille, Annelore Cortoos, Anil Londhe, Panagiotis Mavros, Thomas S Lin, Saad Z Usmani

**Affiliations:** University of Chicago Medical Center, Chicago, IL, USA; Department of Internal Medicine, Division of Hematology, Mayo Clinic, Rochester, MN, USA; Janssen Scientific Affairs, LLC, Horsham, PA, USA; Janssen Research & Development, Raritan, NJ, USA; Analysis Group, Inc., Montreal, QC, Canada; Janssen Scientific Affairs, LLC, Horsham, PA, USA; Janssen Global Services, LLC, Raritan, NJ, USA; Analysis Group, Inc., Montreal, QC, Canada; Janssen Scientific Affairs, LLC, Horsham, PA, USA; Janssen Research & Development, Raritan, NJ, USA; Janssen Scientific Affairs, LLC, Horsham, PA, USA; Janssen Scientific Affairs, LLC, Horsham, PA, USA; Levine Cancer Institute, Atrium Health, Charlotte, NC, USA

**Keywords:** multiple myeloma, daratumumab, progression-free survival, cytogenetics, minimal residual disease

## Abstract

**Background:**

Patients with high-risk, newly diagnosed multiple myeloma (HR-NDMM) who are ineligible for autologous stem cell transplant (ASCT) have limited first-line treatment options. Recent meta-analyses evaluating the impact of incorporating daratumumab in the backbone regimen on progression-free survival (PFS) have found mixed results in these patients.

**Materials and Methods:**

A pooled analysis of patient-level data for ASCT-ineligible patients with HR-NDMM [ie, del(17p), t(4;14), t(14;16)] from the MAIA and ALCYONE trials; stratified by study identifier and adjusting for cytogenetic abnormality subtype, baseline performance status, International Staging System stage, myeloma type, and renal impairment; was conducted. Impact of daratumumab on PFS and rates of complete response or better (≥CR), minimal residual disease (MRD)-negative CR, very good partial response or better (≥VGPR), and overall response (ORR) was compared to control.

**Results:**

Among 101 patients in the daratumumab and 89 patients in the control cohort, median follow-up was 43.7 months. Daratumumab reduced the risk of progression or death by 41% (adjusted hazard ratio for PFS [95% confidence interval (CI)] = 0.59 [0.41-0.85]) versus control. At 36 months, the estimated proportion of patients who did not progress and were still alive was 41.3% in the daratumumab and 19.9% in the control cohort. Rates of ≥CR (41.6% vs. 22.5%), MRD-negative CR (24.8% vs. 5.6%), ≥VGPR (75.2% vs. 46.1%), and ORR (92.1% vs. 74.2%) were higher for daratumumab versus control.

**Conclusion:**

These findings demonstrate that incorporation of daratumumab in frontline treatment regimens reduced the risk of progression or death and improved response rates among ASCT-ineligible HR-NDMM patients.

## Teaser

Using a pooled analysis of patient-level data from the MAIA and ALCYONE clinical trials, this study provides an updated analysis of the effect of daratumumab in cytogenetically high-risk newly diagnosed multiple myeloma.

**Implications for Practice**

Patients with high-risk, newly diagnosed multiple myeloma (HR-NDMM) who are ineligible for autologous stem cell transplant have limited treatment options. Using a stratified pooled analysis of patient-level data from MAIA and ALCYONE, this study found that the incorporation of daratumumab into first-line treatment regimen reduced the risk of progression or death by 41%, increased the rate of CR or better by nearly 2-fold, and increased the rate of MRD-negative CR by more than 4-fold among patients with HR-NDMM. These findings provide additional evidence supporting the use of daratumumab-based first-line treatments in this high-risk population of patients.

## Introduction

Multiple myeloma (MM) is characterized by the accumulation of neoplastic plasma cells in the bone marrow and production of monoclonal M protein detectable in the urine and blood.^1^ MM is uncommon, but remains the second most common hematologic cancer, accounting for approximately 10% of all hematologic cancers.^1,2^ In 2020, the incidence of MM was expected to be 32,270, and the number of MM-related deaths was expected to be 12,830 in the US (US).^3^ With the introduction of several therapeutic options, survival in MM has improved over the past few decades.^4,5^ However, despite numerous treatment options and the use of a multidrug strategy to improve patient outcomes, MM remains largely incurable and there is considerable variation in survival.^5^ Notably, patients with a high-risk cytogenetic profile [eg, del(17p), t(4;14), or t(14;16)] have more aggressive disease than those with standard-risk cytogenetic features.^6-9^ In addition, patients with high-risk cytogenetic profiles who are ineligible for autologous stem cell transplant (ASCT) have limited first-line treatment options that offer consistent and sustained improvements in outcomes.^10^ Since remission duration decreases with each relapse, selecting effective frontline treatments for patients with newly diagnosed MM (NDMM), especially elderly patients who may not have the opportunity to receive later lines of therapy,^11^ is necessary to optimize patient outcomes.^5,12^

Among patients with NDMM who are ineligible for ASCT, daratumumab has been approved in combination with lenalidomide and dexamethasone (D-Rd) and in combination with bortezomib, melphalan, and prednisone (D-VMP), based on 2 randomized phase III clinical trials, MAIA (NCT02252172) and ALCYONE (NCT02195479), respectively. These trials evaluated the effect of daratumumab combination therapies on progression-free survival (PFS), rates of response and minimal residual disease (MRD) negativity, and overall survival (OS).^13,14^ Together, these 2 trials included 190 (15%) patients with high-risk cytogenetic profiles. The individual studies were not powered to detect a difference in PFS in patients with high-risk cytogenetics; however, in each trial, incorporation of daratumumab in treatment regimens was associated with a nonstatistically significant reduction in risk of progression or death in high-risk patients.^13-15^ A recent meta-analysis by Chong et al of the ASCT-ineligible patients with high-risk NDMM from the MAIA (D-Rd vs. Rd; median follow-up of 28.0 months^13^) and ALCYONE (D-VMP vs. VMP; median follow-up of 16.5 months^14^) trials also found that incorporation of daratumumab was associated with a nonstatistically significant improvement in PFS.^16^ Another meta-analysis by Premkumar et al pooling these 2 trials (same data cuts as Chong et al) with the ASCT-eligible population from the CASSIOPEIA trial (NCT02541383; daratumumab, bortezomib, thalidomide, and dexamethasone [D-VTd] vs. VTd; median follow-up of 18.8 months^17^) found a similar nonstatistically significant improvement in PFS with the addition of daratumumab.^18^ However, when using more recent data with longer follow-up from the MAIA trial (median follow-up of 36.4 months^19^) and combining data from MAIA, ALCYONE and CASSIOPEIA, Giri et al demonstrated that incorporation of daratumumab was associated with significantly improved PFS in patients with high-risk NDMM.^20^

Using a stratified analysis of patient-level data from the MAIA^21^ and ALCYONE^15^ clinical trials, the current study aimed to provide an updated and more robust analysis of the effect of daratumumab in cytogenetically high-risk NDMM. Specifically, this study focused on a homogenous population of ASCT-ineligible patients and incorporated longer follow-up data than previous meta-analyses. Additionally, it adjusted for patient-level imbalances in baseline characteristics between the daratumumab and control cohorts, and evaluated additional endpoints, including response endpoints (eg, overall response rate [ORR], complete response [CR] or better, very good partial response [VGPR] or better) and MRD-negative CR.

## Materials and Methods

### Data Source and Study Design

A stratified pooled analysis of patient-level data was performed for patients with baseline high-risk cytogenetics [ie, del(17p), t(4;14), or t(14;16), as defined by both International Myeloma Working Group (IMWG) consensus criteria and study protocols, identified through fluorescence in situ hybridization (FISH) or karyotype tests] from 2 randomized clinical trials, MAIA (data cut from the 2020 American Society of Hematology [ASH] publication; median follow-up of 47.9 months)^21^ and ALCYONE (data cut from the 2020 *Lancet* publication; median follow-up of 40.1 months).^15^ These 2 trials evaluated daratumumab combination therapies versus control in ASCT-ineligible patients, with MAIA evaluating D-Rd versus Rd^21^ and ALCYONE evaluating D-VMP versus VMP.^15^ Study designs of these 2 studies were published previously.^13,14^

### Outcomes

The primary endpoint was PFS, defined as the time from randomization to progressive disease or death, assessed by a computerized algorithm according to IMWG response criteria. Patients who did not have an event were censored at the date of last disease assessment, prior to initiation of subsequent antimyeloma therapy, withdrawal of consent, loss to follow up, or last date of participation in the study, whichever came first.

Secondary endpoints included best response and MRD-negative CR, evaluated at any time point between randomization and disease progression or start of subsequent therapy, whichever occurred first. Response was measured using ORR, defined as the proportion of patients who achieved partial response (PR) or better (ie, stringent complete response [sCR], CR, VGPR, or PR). The proportions of patients with VGPR or better, and with CR or better, were also evaluated. MRD-negative CR was defined as the proportion of patients who had a best response of CR or better and MRD-negative status assessed by next-generation sequencing using a threshold of 1 tumor cell per 10^5^ white cells.

### Statistical Analysis

The balance in baseline characteristics between the daratumumab and control cohorts was evaluated in each trial separately, using standardized differences (std diff), a measure of effect size independent of sample size,^22^ where characteristics with a std diff < 10% were considered balanced.^23^

Multivariable stratified Cox regression analysis based on the combined data was used to assess the impact of daratumumab compared to control treatment on PFS, with the study identifier (ie, MAIA or ALCYONE) as the stratification factor and adjusting for baseline characteristics that were not balanced between cohorts within each trial and that were deemed to be clinically relevant (ie, type of cytogenetic abnormalities [ie, del(17p), t(4; 14), t(14; 16)], Eastern Cooperative Oncology Group [ECOG] performance status, International Staging System [ISS] stage, type of MM [ie, IgG vs. non-IgG], and renal impairment [defined as creatinine clearance <60 mL/minute]). Cox regression analyses for PFS were also performed for each trial separately, as well as for the pooled subgroup of patients with del(17p), the most common type of cytogenetic abnormality in these patients. Additionally, Kaplan-Meier curves and estimates were reported for the pooled population.

The proportion of patients reaching best response of CR or better, MRD-negative CR, best response of VGPR or better, and ORR was compared between the 2 cohorts using stratified logistic regression, with the study identifier as the stratification factor, adjusting for baseline characteristics. Unadjusted relative response ratio (RR; ratio of probability of response in daratumumab cohort to the probability of response in control cohort, also known as *relative risk*) was calculated using the Mantel-Haenszel method, with the study identifier as the stratification factor.

A sensitivity analysis additionally controlling for age (<75 vs. ≥75 years) was conducted for all outcomes.

## Results

### Baseline Characteristics and Treatment Duration

The baseline characteristics of the daratumumab (*N* = 101) and the control cohorts (*N* = 89) for ASCT-ineligible patients with NDMM and high-risk cytogenetic profiles were evaluated for each trial separately (Table 1). Median age was 74.0 and 74.5 years in MAIA and 70.0 and 71.0 years in ALCYONE, for the daratumumab and the control cohorts, respectively. Baseline characteristics were generally similar between the daratumumab and control cohorts, but some differences between the cohorts were noted (Table 1). In MAIA, baseline characteristics with a std diff ≥10% included type of MM, ISS stage, del(17p) and t(4;14), and renal impairment, and thus were considered imbalanced. In ALCYONE, baseline characteristics with a std diff ≥10% included age ≥75 years, baseline ECOG score, and t(4;14) translocation, and thus were considered imbalanced. The median duration of treatment was 19.9 months in the daratumumab group and 12.0 months in the control group.

**Table 1.** Demographic and baseline characteristics of ASCT-ineligible high-risk NDMM patients in MAIA and ALCYONE

	MAIA			ALCYONE		
	Daratumumab + control	Control	Std diff^a^	Daratumumab + control	Control	Std diff^a^
	*N* = 48	*N* = 44		*N* = 53	*N* = 45	
Age, years						
Median	74.5	74.0		71.0	70.0	
Category, *n* (%)						
<75	24 (50.0%)	24 (54.5%)	9.1%	34 (64.2%)	34 (75.6%)	25.0%
≥75	24 (50.0%)	20 (45.5%)	9.1%	19 (35.8%)	11 (24.4%)	25.0%
Male, *n* (%)	24 (50.0%)	20 (45.5%)	9.1%	25 (47.2%)	20 (44.4%)	5.5%
ECOG score, *n* (%)						
0	17 (35.4%)	18 (40.9%)	11.3%	10 (18.9%)	13 (28.9%)	23.7%
1	18 (37.5%)	17 (38.6%)	2.3%	24 (45.3%)	23 (51.1%)	11.7%
≥2^b^	13 (27.1%)	9 (20.5%)	15.6%	19 (35.8%)	9 (20.0%)	35.9%
Type of MM by immunofixation or serum FLC, *n* (%)						
IgG	35 (72.9%)	28 (63.6%)	20.0%	27 (50.9%)	25 (55.6%)	9.3%
Non-IgG	13 (27.1%)	16 (36.4%)	20.0%	26 (49.1%)	20 (44.4%)	9.3%
ISS stage^c^, *n* (%)						
I	6 (12.5%)	8 (18.2%)	15.8%	6 (11.3%)	4 (8.9%)	8.1%
II	21 (43.8%)	15 (34.1%)	19.9%	23 (43.4%)	18 (40.0%)	6.9%
III	21 (43.8%)	21 (47.7%)	8.0%	24 (45.3%)	23 (51.1%)	11.7%
Cytogenetic risk^d^, *n* (%)						
del(17p)	25 (52.1%)	29 (65.9%)	28.4%	29 (54.7%)	27 (60.0%)	10.7%
t(4;14)	21 (43.8%)	12 (27.3%)	35.0%	25 (47.2%)	17 (37.8%)	19.1%
t(14;16)	4 (8.3%)	5 (11.4%)	10.2%	6 (11.3%)	6 (13.3%)	6.1%
Renal impairment^e^, *n* (%)	25 (52.1%)	19 (43.2%)	17.9%	22 (41.5%)	17 (37.8%)	7.6%

Std diff is a measure of effect size independent of sample size,^22^ where characteristics with a std diff < 10% were considered balanced.^23^

ALCYONE had maximum baseline ECOG score of 2.

ISS staging was derived based on the combination of serum β2-microglobulin and albumin.

Cytogenetic risk was based on FISH or karyotype testing.

Renal impairment was defined as having baseline creatinine clearance less than 60 mL/minute.

Abbreviations: ASCT, autologous stem cell transplant; ECOG, Eastern Cooperative Oncology Group; FLC, free light chain; ISS, International Staging System; MM, multiple myeloma; NDMM, newly diagnosed multiple myeloma; Std diff, standardized difference.

### Progression-free Survival

**Figure 1.**
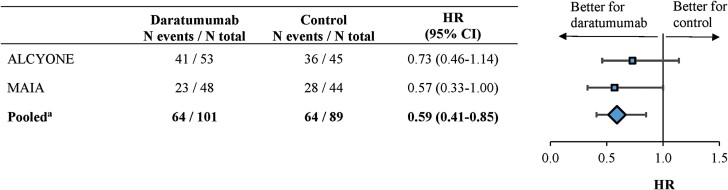
Forest plot of PFS among ASCT-ineligible patients with high-risk NDMM from MAIA and ALCYONE, separately and pooled. Abbreviations: ASCT, autologous stem cell transplant; CI, confidence interval; HR, hazard ratio; NDMM, newly diagnosed multiple myeloma; PFS, progression-free survival. ^a^For the pooled analysis, a multivariate stratified Cox regression analysis was used to calculate adjusted HR, with the study identifier as the stratification factor. HR was adjusted for cytogenetic abnormalities [ie, del(17p), t(4, 14), 4(14, 16)], baseline Eastern Cooperative Oncology Group performance status, International Staging System stage, type of multiple myeloma (ie, IgG vs. non-IgG), and renal impairment (defined as creatinine clearance <60 mL/minute).

**Figure 2.**
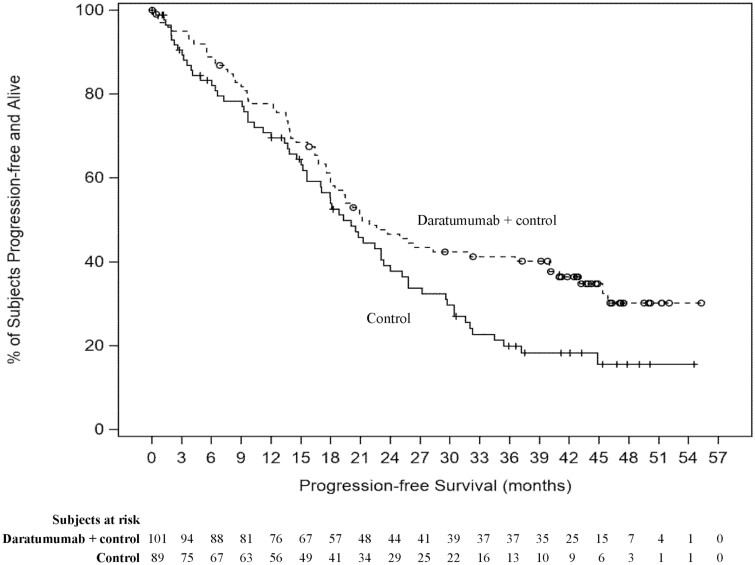
Kaplan-Meier curves of PFS among ASCT-ineligible patients with high-risk NDMM pooled from MAIA and ALCYONE. Abbreviations: ASCT, autologous stem cell transplant; NDMM, newly diagnosed multiple myeloma; PFS, progression-free survival.

Pooling these 2 trials together and using the most recent follow-up data (median follow-up of 43.7 months), the adjusted HR for PFS (95% confidence interval [CI]) among ASCT-ineligible patients with high-risk NDMM was 0.59 (0.41-0.85) (*P* = .0046; Fig. 1), a 41% reduction in the risk of disease progression or death for the daratumumab cohort compared to the control cohort. In the individual trials, the adjusted HR for PFS was 0.73 (0.46-1.14) in ALCYONE and 0.57 (0.33-1.00) in MAIA (Fig. 1). A sensitivity analysis additionally controlling for age differences for the pooled data yielded similar results (adjusted HR [95% CI] = 0.59 [0.41-0.85]; *P* = .0044). Also, among the pooled subgroup of patients with del(17p) at baseline (N daratumumab: 54 patients; N control: 56 patients), the adjusted HR for PFS was 0.63 (0.39-1.03) (*P* = .0659). Fig. 2 presents the Kaplan-Meier curves of PFS among the overall pooled ASCT-ineligible patients with high-risk NDMM. The median PFS was 21.2 months in the daratumumab cohort and 19.3 months in the control cohort, and the proportion of patients who did not progress and were still alive at 36 months was 41.3% in the daratumumab cohort and 19.9% in the control cohort.

### Response

The proportion of patients with CR or better was nearly 2-fold higher in the daratumumab cohort compared to the control cohort (41.6% vs. 22.5%; RR [95% CI] = 1.85 [1.18-2.90]; Table 2). The median time to CR or better was 9.3 months (range: 3.5-34.5) in the daratumumab cohort and 7.1 months (range: 2.3-43.8) in the control cohort. The rate of MRD-negative CR, using a threshold of 1 tumor cell per 10^5^ cells, was more than 4-fold higher among patients in the daratumumab cohort compared to the control cohort (24.8% vs. 5.6%; RR [95% CI] = 4.35 [1.75-10.82]; Table 2). Similar findings were observed for the proportion of patients with best response of VGPR or better (75.2% vs. 46.1%; RR [95% CI] = 1.64 [1.27-2.10]) and for the ORR (92.1% vs. 74.2%; RR [95% CI] = 1.24 [1.08-1.42]; Table 2).  Adjusted odds ratios, including the sensitivity analysis additionally controlling for age differences, yielded similar results and indicated a significantly higher likelihood of response for the daratumumab cohort when compared to the control cohort (Table 2).

**Table 2.** Response rates and MRD-negative CR rates among ASCT-ineligible high-risk NDMM patients from MAIA and ALCYONE

	Daratumumab + control	Control	Relative response ratio^a^(95% CI)	Adjusted OR^b^(95% CI)	*P*-value	Sensitivity analysis adjusting for age	
	*N* = 101	*N* = 89				Adjusted OR^c^(95% CI)	*P*-value
Best response							
CR or better (sCR + CR)	42 (41.6%)	20 (22.5%)	1.85 (1.18-2.90)	2.63 (1.34-5.16)	0.0051	2.57 (1.30-5.06)	.0064
sCR	27 (26.7%)	5 (5.6%)	–	–	–	–	–
CR	15 (14.9%)	15 (16.9%)	–	–	–	–	–
MRD-negative CR	25 (24.8%)	5 (5.6%)	4.35 (1.75-10.82)	5.50 (1.97-15.34)	0.0011	5.31 (1.89-14.88)	.0015
VGPR	34 (33.7%)	21 (23.6%)	–	–	–	–	–
PR	17 (16.8%)	25 (28.1%)	–	–	–	–	–
SD	3 (3.0%)	19 (21.3%)	–	–	–	–	–
PD	0 (0.0%)	0 (0.0%)	–	–	–	–	–
NE	5 (5.0%)	4 (4.5%)	–	–	–	–	–
VGPR or better (sCR + CR + VGPR)	76 (75.2%)	41 (46.1%)	1.64 (1.27-2.10)	4.03 (2.09-7.78)	<0.0001	4.08 (2.10-7.91)	<.0001
Overall response (sCR + CR + VGPR + PR)	93 (92.1%)	66 (74.2%)	1.24 (1.08-1.42)	4.88 (1.94-12.27)	0.0008	4.71 (1.87-11.88)	.0010

Relative response ratio was calculated using the Mantel–Haenszel method, with the study identifier as the stratification factor.

Adjusted OR was calculated using stratified logistic regression analysis, with the study identifier as the stratification factor. OR was adjusted for cytogenetic abnormalities [ie, del(17p), t(4, 14), 4(14, 16)], baseline Eastern Cooperative Oncology Group performance status, International Staging System stage, type of multiple myeloma (ie, IgG vs. non-IgG), and renal impairment (defined as creatinine clearance <60 mL/minute).

OR was additionally adjusted for age (<75 vs. ≥75 years).

Abbreviations: ASCT, autologous stem cell transplant; CI, confidence interval; CR, complete response; MRD, minimal residual disease; NDMM, newly diagnosed multiple myeloma; NE, not evaluable; OR, odds ratio; PD, progressive disease; PR, partial response; sCR, stringent complete response; SD, stable disease; VGPR, very good partial response.

## Discussion

This analysis of ASCT-ineligible patients with high-risk NDMM from the MAIA and ALCYONE clinical trials demonstrated that the incorporation of daratumumab in first-line treatment regimens was associated with improved PFS and response rates, including CR or better, MRD-negative CR, and VGPR or better, in this patient population. After adjustment for differences in baseline characteristics, there was a 41% reduction in the risk of disease progression or death when daratumumab was incorporated into first-line treatment. The consistency of the results across all evaluated efficacy endpoints and sensitivity analyses demonstrates the robustness of the findings and the benefit of daratumumab in the ASCT-ineligible high-risk NDMM population.

Previous meta-analyses evaluating the effect of daratumumab in patients with high-risk NDMM found mixed results, and Chong et al recognized that the data available to them at the time of their study was insufficient to demonstrate a clear benefit of daratumumab in the ASCT-ineligible NDMM population.^16,18,20^ The present pooled analysis fills this gap by addressing some important concerns of the previous studies. First, previous meta-analyses evaluating the effect of daratumumab in patients with high-risk NDMM had shorter follow-up than the current study for the MAIA (Giri et al: median follow-up of 36.4 months^19^; Premkumar et al and Chong et al: median follow-up of 28.0 months^13^) and the ALCYONE (Giri et al, Premkumar et al, and Chong et al: median follow-up of 16.5 months^14^) trials.^16,18,20^ Of note, while a meta-analysis by Wang et al using the most up-to-date ALCYONE data (median follow-up of 40.1 months^15^) found that incorporating daratumumab among patients with high-risk cytogenetics was associated with a better ORR than control, the meta-analysis also included patients with relapsed or refractory MM from the POLLUX (NCT02076009; D-Rd vs. Rd) and CASTOR trials (NCT02136134; daratumumab, bortezomib, and dexamethasone [D-Vd] vs. Vd), and is thus not as relevant, since it did not separately present the effect for patients with high-risk NDMM.^24^ The current study used the latest data cut with longer median follow-up for both the MAIA (data cut from the 2020 American Society of Hematology [ASH] publication; median follow-up of 47.9 months)^21^ and ALCYONE trials (data cut from the 2020 *Lancet* publication; median follow-up of 40.1 months).^15^ Second, as opposed to Giri et al and Premkumar et al,^18,20^ the current study focused on a more homogeneous population of patients with high-risk NDMM (ie, ASCT-ineligible patients) and did not include the CASSIOPEIA study, which was conducted in ASCT-eligible patients and defined cytogenetically high-risk disease differently.^17^ Finally, the current study applied a more robust methodology by using patient-level data to further adjust for patient-level imbalances in baseline characteristics between the daratumumab and control cohorts, and by using stratified analyses to control for structural differences between trials, notably related to the uneven follow-up times and the different control regimens in MAIA and ALCYONE. Pooling data from the 2 trials and leveraging the most recent data from each trial helped alleviate previous uncertainties around the impact of daratumumab among patients with high-risk NDMM.

As mentioned previously, using longer follow up times from the trials helped demonstrate a significant benefit in the incorporation of daratumumab in first-line treatment regimens in this population. Indeed, the descriptive Kaplan-Meier curves show that the PFS rates remained relatively similar between the 2 cohorts until approximately 18 months, and then diverged with a substantial and clinically significant difference at later time points (eg, the estimated PFS rates at 36 months: 41.3% vs. 19.9% in the daratumumab and control cohorts, respectively; Fig. 2). We hypothesize that the similarity in the earlier portion of the curve may be due to more rapid progression of patients who failed to attain CR in both cohorts, and that the separation of the PFS curves at later time points may be driven by the difference in the rates of CR or better across cohorts. Indeed, the proportion of patients who attained CR or better was 41.6% for the daratumumab cohort compared to only 22.5% for the control cohort. Similarly, the rate of MRD-negative CR was more than 4-fold higher among patients in the daratumumab cohort (24.8%) compared to the control cohort (5.6%). Previous studies have shown CR and/or MRD negativity to be associated with improved PFS and other survival outcomes among patients with NDMM, highlighting the clinical importance of striving for a CR or better response in this patient population.^25-28^ In addition, a prior study has shown that depth of response to daratumumab increases over time.^29^ With daratumumab being given until progression in both MAIA and ALCYONE, and the median time to CR being 9.3 months, the findings of the current study suggest the importance of longer duration of therapy to maximize depth of response, especially in this high-risk patient population. When exploring PFS by response status through a descriptive analysis, median PFS was longer for the daratumumab cohort relative to the control cohort among patients achieving a best response of CR or better (median PFS: not reached vs. 31 months), while median PFS was similar in both cohorts for those who did not achieve CR (median PFS: 16.4 for the daratumumab cohort; 15.6 months for the control cohort; Supplementary Fig. S1). However, further studies using appropriate statistical methods are warranted to fully understand the association between CR and PFS in this population.

The findings of this study have important clinical implications. High-risk NDMM represents an aggressive condition in which durable responses are difficult to achieve.^30^ The current study demonstrates that the incorporation of daratumumab in the first-line treatment regimen doubles the likelihood of achieving CR or better and increases the rate of MRD-negative CR more than 4-fold. Of note, the likelihood of achieving CR or better was similar in the pooled high-risk patients of this analysis (2.63 [1.34-5.16]; Table 2) compared to the standard-risk subgroup of MAIA (odds ratio [95% CI] = 2.54 [1.80, 3.61]; data on file) and ALCYONE (2.70 [1.86-3.91]; data on file). The incorporation of daratumumab in the treatment regimen among patients with high-risk NDMM resulted in a significant PFS benefit versus control (HR [95% CI] =0.59 [0.41-0.85];  Fig. 1), but numerically less pronounced than among the patients with standard-risk NDMM in MAIA and ALCYONE (MAIA: HR [95% CI] = 0.48 [0.38-0.62]; ALCYONE: 0.36 [0.28-0.45]; data on file). While the current study did not evaluate specifically the relationship between the depth of response and the likelihood of achieving optimal long-term treatment outcomes, it provided additional evidence that incorporation of daratumumab into the first-line treatment regimen benefits patients with high-risk NDMM both in terms of response and PFS.

This pooled analysis and the Giri et al meta-analysis are the initial 2 studies showing a benefit for the addition of monoclonal antibody to backbone therapy for high-risk MM patients,^20^ as elotuzumab did not improve outcomes when added to bortezomib, lenalidomide, and dexamethasone backbone therapy in the SWOG-1211 trial.^31^ Other trials evaluating the addition of daratumumab to various control regimens among ASCT-ineligible patients with NDMM are currently ongoing, including CEPHEUS (NCT03652064) and GEM2017FIT (NCT03742297).

### Limitations

The data analyzed for this study are limited to the information collected in the trials; therefore, additional extraneous factors which may impact results may not be captured. Relatedly, OS was not reported as part of the current study, given that the data collected in the trials was not sufficiently mature at the time of the current analysis. Since all included participants were required to meet a fixed set of inclusion criteria for enrollment in the study, results of this study may not be representative of the general high-risk NDMM ASCT-eligible population. Lastly, since this analysis was post hoc, statistical significance for the outcomes could not be concluded.

## Conclusions

In a combined analysis of clinical trial data from MAIA and ALCYONE among transplant-ineligible, patients with high-risk NDMM, the incorporation of daratumumab into first-line treatment regimens reduced the risk of progression or death by 41%, increased the rate of CR or better by nearly 2-fold, and increased the rate of MRD-negative CR by more than 4-fold among ASCT-ineligible patients with high-risk NDMM. These findings provide additional evidence supporting the use of daratumumab-based treatment in this high-risk population of patients with a particularly high unmet need.

## Supplementary Material

oyac067_suppl_Supplementary_Material

## Data Availability

The patient-level data used in this study are available from the MAIA and ALCYONE publications. The data sharing policy of Janssen Pharmaceutical Companies of Johnson & Johnson is available at https://www.janssen.com/clinical-trials/transparency. As noted on this website, requests for access to the study data can be submitted through the Yale Open Data Access (YODA) Project, at http://yoda.yale.edu.
