# Supplementary material for: Daratumumab Improves Depth of Response and Progression-free Survival in Transplant-ineligible, High-risk, Newly Diagnosed Multiple Myeloma
Source: Oncologist. 2022 Apr 24;27(7):e589–96. doi: 10.1093/oncolo/oyac067 (PMC9256027; doi:10.1093/oncolo/oyac067)

Supplemental Figure 1. PFS among ASCT-ineligible high-risk NDMM patients from MAIA and ALCYONE, stratified by level of response

ASCT = autologous stem cell transplant; CI = confidence interval; CR = complete response; HR = hazard ratio; NDMM = newly diagnosed multiple myeloma; PFS = progression-free survival.


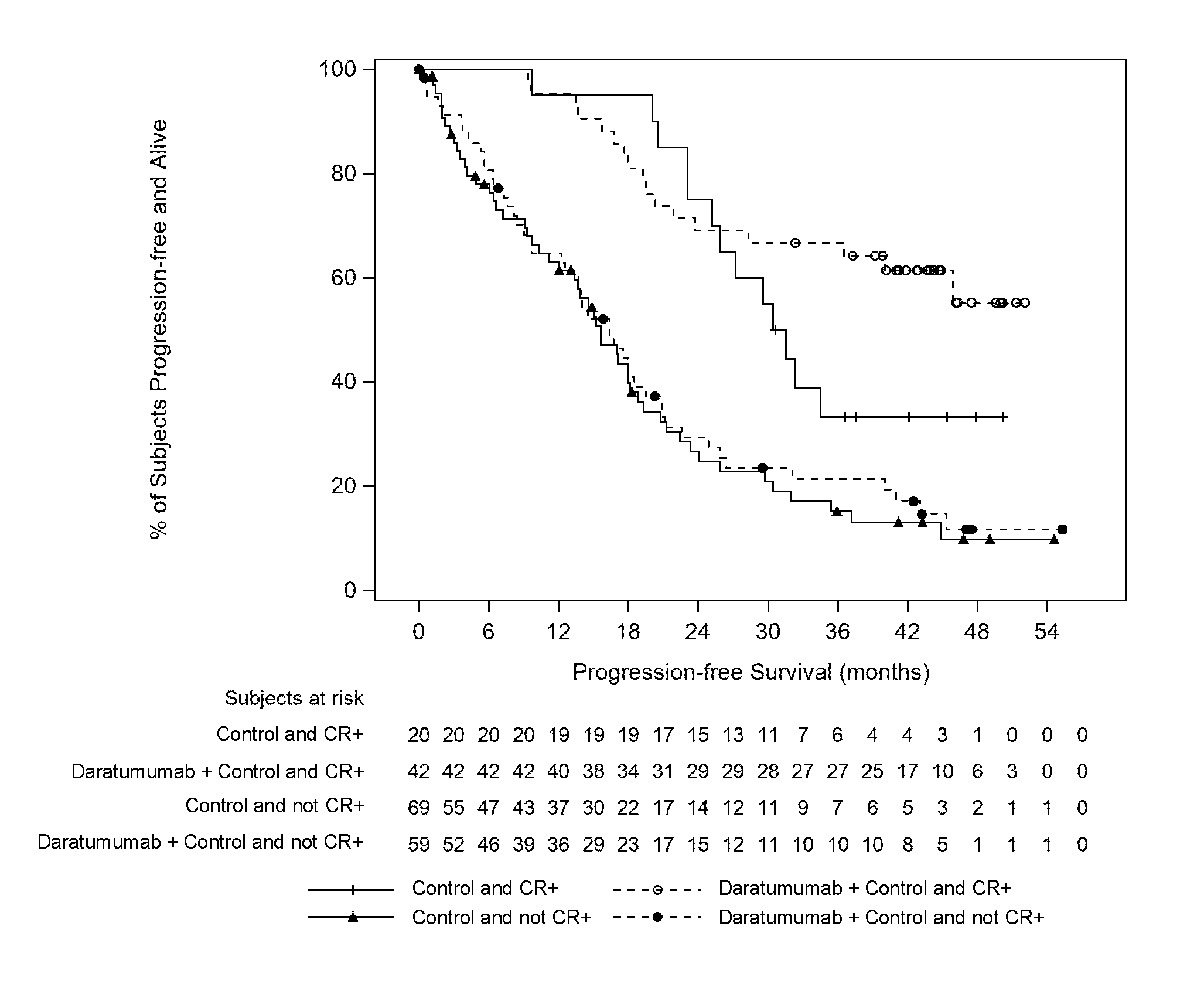

Supplement: oyac067_suppl_Supplementary_Material [file oyac067_suppl_supplementary_material.docx]
